# A Case of Cornelia de Lange Syndrome: Difficulty in Prenatal Diagnosis

**DOI:** 10.1155/2019/4530491

**Published:** 2019-05-13

**Authors:** Tadatsugu Kinjo, Keiko Mekaru, Miyuki Nakada, Hayase Nitta, Hitoshi Masamoto, Yoichi Aoki

**Affiliations:** Department of Obstetrics and Gynecology, Graduate School of Medicine, University of the Ryukyus, 207 Uehara Nishihara, Okinawa 903-0215, Japan

## Abstract

We report a case of Cornelia de Lange syndrome (CdLS) where prenatal diagnosis was not made even with major anomaly. A 33-year-old Japanese woman was referred to our institution at 23 weeks of gestation because of fetal forearm defect. Ultrasound examination revealed short forearms and short humeri and femurs (–2.1 SD). The fetal estimated body weight was 450 g (–1.3 SD). Fetal MRI at 26 weeks of gestation revealed short forearms and hypoplasty of hand fingers. Fetal growth restriction became evident thereafter, leading to intrauterine fetal death occurring at 29 weeks of gestation. A stillbirth baby was of 798 g in body weight and 33.0 cm in length. External examination showed a low hairline, synophrys, low-set ear, hypertrichosis, and smooth long philtrum with thin lips. The neck appeared short and broad. Finally, CdLS was diagnosed. The prenatal diagnosis might be possible as the arm findings were totally characteristic in a small fetus, regardless of whether an overhanging upper lip was identified. Because CdLS is a rare condition, it is important to consider its possibility as a part of differential diagnosis.

## 1. Introduction

The Cornelia de Lange syndrome (CdLS) was first reported by Vrolik in 1849 and Brachmann in 1916, followed by Cornelia de Lange in 1933, after whom the syndrome is named [1–3]. CdLS is a clinically variable disorder mainly characterized by distinctive facial features, growth restriction, hirsutism, psychomotor delay, intellectual disability, and malformations of the upper limbs [4]. The precise prevalence of the disease is unknown but is estimated to be 1–10:100,000 [5]. The diagnosis of CdLS is generally performed after birth, but the syndrome could be suspected during the second and third trimester of pregnancy by observing a wide range of features through ultrasound examination. However, a previous report showed that a routine prenatal ultrasonography failed to detect more than two-thirds of cases of CdLS with major malformations [5].

We report a case of CdLS where prenatal diagnosis was not made even with major anomaly.

## 2. Case Presentation

**Figure 1:**
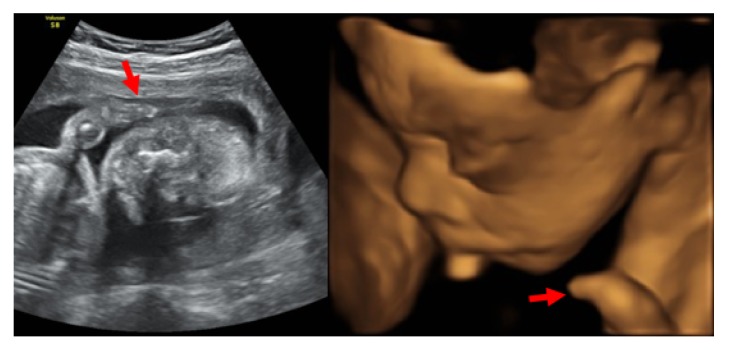
Two-dimensional (right) and three-dimensional (left) prenatal ultrasound images showing short forearms of 7 mm and 9 mm (arrows) and short humeri and femurs (–2.1 standard deviation, SD).

**Figure 2:**
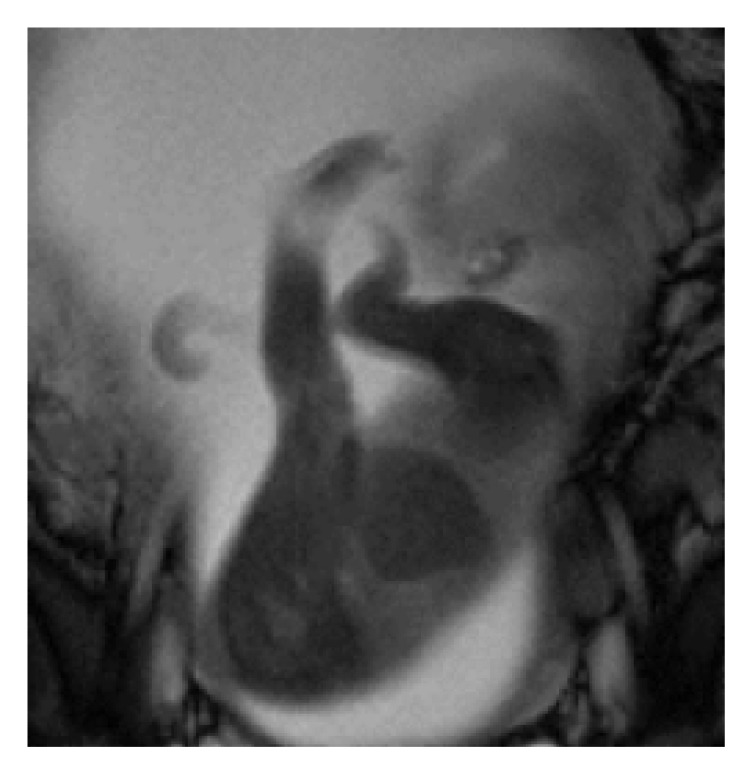
Fetal MRI at 26 weeks of gestation showing short forearms and hypoplasty of hand fingers.

**Figure 3:**
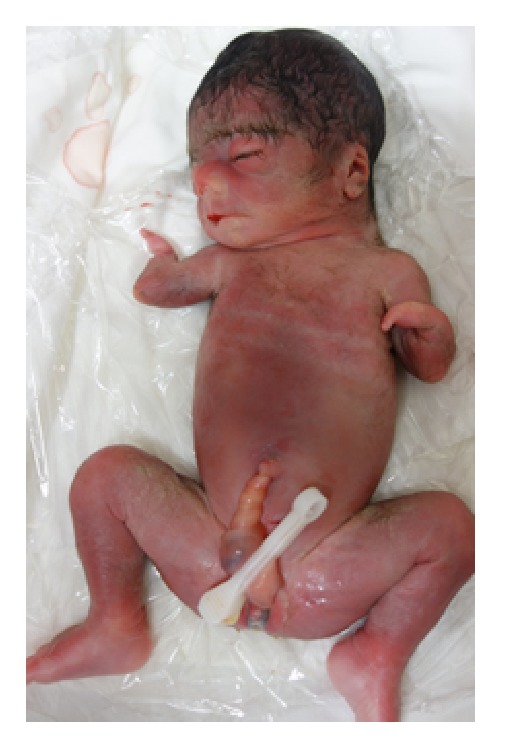
External examination showing a low hairline, synophrys, low-set ear, hypertrichosis, and smooth long philtrum with thin lips. The neck appears short and broad.

A 33-year-old Japanese woman, para 0-0-1-0, with no known family and past histories of the disease, was referred to our institution at 23 weeks of gestation because of fetal forearm defect as detected by ultrasound examination at 21 weeks and 5 days of gestation. Ultrasound examination (Figure 1) revealed short forearms of 7 mm and 9 mm and short humeri and femurs (–2.1 standard deviation, SD). The fetal estimated body weight was 450 g (–1.3 SD). Fetal MRI at 26 weeks of gestation revealed short forearms and hypoplasty of hand fingers (Figure 2). The serum analyses of the mother showed no TORCH syndrome, and the exposure of drugs as teratogen was denied. No other anomalies were found. However, fetal growth restriction (FGR) became evident thereafter, leading to intrauterine fetal death (IUFD) occurring at 29 weeks of gestation. A stillbirth baby was of 798 g in body weight and 33.0 cm in length. External examination showed a low hairline, synophrys, low-set ear, hypertrichosis, and smooth long philtrum with thin lips (Figure 3). The neck appeared short and broad. Finally, CdLS was diagnosed. Autopsy and genetic and chromosomal analyses were declined.

## 3. Discussion

Because of a wide range of features of CdLS, its diagnosis is generally performed after birth, although it could be suspected by ultrasound examination during the second and third trimester of pregnancy. Indeed, CdLS diagnosis is still underperformed and extremely rare before birth. Avagliano et al. [6] proposed that following a sequence of detailed scans and examinations, CdLS-affected fetuses could be diagnosed in utero, when one or more characteristics, such as FGR, limb defects, facial abnormalities, diaphragmatic hernia, and heart diseases, are detected and confirmed by specific molecular diagnostic tests, such as Nipped-B-like protein (NIPBL), structural maintenance of chromosomes 1A (SMC1A), structural maintenance of chromosomes 3 (SMC3), human homolog of Schizosaccharomyces pombe radiation sensitive mutant 21 (RAD21), and histone deacetylase 8 (HDAC8) [7]. To date, maternal serum pregnancy associated placental protein-A (PAPP-A), which is a glycoprotein produced by the placenta, level is reported to be a reliable predictor of CdLS in the first and second trimester of pregnancy. It is not considered to be a specific marker, but a very low PAPP-A can also support this diagnosis [6]. PAPP-A was not done in our patient, because it is not routinely offered in Japan.

Although FGR is nearly always detected as was in our case, it is a nonspecific characteristic; therefore, other associated ultrasound findings should be detected for a more appropriate prenatal prediction of the syndrome [8]. Limb abnormalities, ranging from small fingers and hand abnormalities to complete absence of the upper limbs, are often detected in prenatal ultrasonography for CdLS [8]. A careful ultrasound examination of the arms in relation to FGR may lead to the early detection of the possible presence of CdLS. The affected babies present with the following facial characteristics, including a bulging forehead with frontal bossing, depressed nasal bridge, long bulging philtrum, and micrognathia. A three-dimensional and/or two-dimensional ultrasound scan may identify these findings [8, 9]. The prenatal detection associated with limb defects and FGR may significantly improve the early prenatal diagnosis of CdLS. Other malformations, such as diaphragmatic hernia, congenital heart diseases, and anatomical brain disorders, have been reported [6, 8], and ultrasound examination should be performed for a prognostic value.

In our case, FGR and upper limb abnormality were identified but not facial characteristics. Thus, the prenatal diagnosis of CdLS was not made even with major anomaly, but might be possible as the arm findings were totally characteristic in a small fetus, regardless of whether an overhanging upper lip was identified (profile photo not shown). Because CdLS is a rare condition, it is important to consider its possibility as a part of differential diagnosis. Possible diagnostic evidences should lead to tests that could　provide an early diagnosis, at least in some cases, which is important in any rare disease.
